# Supplementary material for: Pyruvate Dehydrogenase Kinase 1 inhibition mediated oxidative phosphorylation enhancement in cartilage promotes osteoarthritis progression
Source: BMC Musculoskelet Disord. 2023 Jul 20;24:597. doi: 10.1186/s12891-023-06585-6 (PMC10357736; doi:10.1186/s12891-023-06585-6)
Supplement: Supplementary file 1 — Additional file 1. [file 12891_2023_6585_MOESM1_ESM.zip › MF.Gsea.1653627316242/GOMF_WIDE_PORE_CHANNEL_ACTIVITY.html]

Details for gene set GOMF\_WIDE\_PORE\_CHANNEL\_ACTIVITY[GSEA]

|  || Dataset | 55457\_collapsed\_to\_symbols.cls.cls #OA\_versus\_normal.cls.cls #OA\_versus\_normal\_repos |
| Phenotype | cls.cls#OA\_versus\_normal\_repos |
| Upregulated in class | normal |
| GeneSet | GOMF\_WIDE\_PORE\_CHANNEL\_ACTIVITY |
| Enrichment Score (ES) | -0.5054668 |
| Normalized Enrichment Score (NES) | -1.5179193 |
| Nominal p-value | 0.055062167 |
| FDR q-value | 0.17736483 |
| FWER p-Value | 1.0 |
Table: GSEA Results Summary

  

Fig 1: Enrichment plot: GOMF\_WIDE\_PORE\_CHANNEL\_ACTIVITY      
 Profile of the Running ES Score & Positions of GeneSet Members on the Rank Ordered List

  

| SYMBOL | TITLE | RANK IN GENE LIST | RANK METRIC SCORE | RUNNING ES | CORE ENRICHMENT || 1 | GJB3 | gap junction protein beta 3 [Source:HGNC Symbol;Acc:HGNC:4285] | 549 | 0.641 | 0.0658 | No |
| 2 | VDAC2 | voltage dependent anion channel 2 [Source:HGNC Symbol;Acc:HGNC:12672] | 2734 | 0.276 | -0.0571 | No |
| 3 | VDAC1 | voltage dependent anion channel 1 [Source:HGNC Symbol;Acc:HGNC:12669] | 3939 | 0.161 | -0.1232 | No |
| 4 | GJB5 | gap junction protein beta 5 [Source:HGNC Symbol;Acc:HGNC:4287] | 5542 | 0.029 | -0.2427 | No |
| 5 | GJD2 | gap junction protein delta 2 [Source:HGNC Symbol;Acc:HGNC:19154] | 6208 | -0.022 | -0.2906 | No |
| 6 | GJA4 | gap junction protein alpha 4 [Source:HGNC Symbol;Acc:HGNC:4278] | 6548 | -0.045 | -0.3093 | No |
| 7 | GJA9 | gap junction protein alpha 9 [Source:HGNC Symbol;Acc:HGNC:19155] | 6689 | -0.055 | -0.3109 | No |
| 8 | PANX1 | pannexin 1 [Source:HGNC Symbol;Acc:HGNC:8599] | 7344 | -0.105 | -0.3438 | No |
| 9 | GJC1 | gap junction protein gamma 1 [Source:HGNC Symbol;Acc:HGNC:4280] | 7350 | -0.106 | -0.3263 | No |
| 10 | GJA3 | gap junction protein alpha 3 [Source:HGNC Symbol;Acc:HGNC:4277] | 7569 | -0.125 | -0.3222 | No |
| 11 | PRF1 | perforin 1 [Source:HGNC Symbol;Acc:HGNC:9360] | 8265 | -0.176 | -0.3464 | No |
| 12 | GJC2 | gap junction protein gamma 2 [Source:HGNC Symbol;Acc:HGNC:17494] | 8516 | -0.195 | -0.3329 | No |
| 13 | GJA1 | gap junction protein alpha 1 [Source:HGNC Symbol;Acc:HGNC:4274] | 9325 | -0.258 | -0.3521 | No |
| 14 | VDAC3 | voltage dependent anion channel 3 [Source:HGNC Symbol;Acc:HGNC:12674] | 9878 | -0.310 | -0.3426 | No |
| 15 | TOMM40 | translocase of outer mitochondrial membrane 40 [Source:HGNC Symbol;Acc:HGNC:18001] | 11977 | -0.589 | -0.4059 | Yes |
| 16 | GJA5 | gap junction protein alpha 5 [Source:HGNC Symbol;Acc:HGNC:4279] | 12095 | -0.619 | -0.3104 | Yes |
| 17 | GJA8 | gap junction protein alpha 8 [Source:HGNC Symbol;Acc:HGNC:4281] | 12787 | -0.949 | -0.2037 | Yes |
| 18 | GJB1 | gap junction protein beta 1 [Source:HGNC Symbol;Acc:HGNC:4283] | 12889 | -1.257 | 0.0009 | Yes |
Table: GSEA details [plain text format]

  

Fig 2: GOMF\_WIDE\_PORE\_CHANNEL\_ACTIVITY      
 Blue-Pink O' Gram in the Space of the Analyzed GeneSet

  

Fig 3: GOMF\_WIDE\_PORE\_CHANNEL\_ACTIVITY: Random ES distribution      
 Gene set null distribution of ES for **GOMF\_WIDE\_PORE\_CHANNEL\_ACTIVITY**
